# Investigation on the Mechanism of Qubi Formula in Treating Psoriasis Based on Network Pharmacology

**DOI:** 10.1155/2020/4683254

**Published:** 2020-06-21

**Authors:** Lin Zhou, Lingyun Zhang, Disheng Tao

**Affiliations:** ^1^Department of Dermatology and Venereal Diseases, Affiliated Hospital of Integrated Traditional Chinese and Western Medicine, Nanjing University of Chinese Medicine, Nanjing 210028, China; ^2^Department of Dermatology and Venereal Diseases, Jiangsu Province Academy of Traditional Chinese Medicine, Nanjing 210028, China; ^3^Nanjing Nuoyu Medical Service Co., Ltd., Jiangning Junhetang Traditional Chinese Medicine Clinic, Nanjing 210000, China

## Abstract

**Objective:**

To elucidate the pharmacological mechanisms of Qubi Formula (QBF), a traditional Chinese medicine (TCM) formula which has been demonstrated as an effective therapy for psoriasis in China.

**Methods:**

The Traditional Chinese Medicine Systems Pharmacology (TCMSP) database, BATMAN-TCM database, and literature search were used to excavate the pharmacologically active ingredients of QBF and to predict the potential targets. Psoriasis-related targets were obtained from Therapeutic Target Database (TTD), DrugBank database (DBD), MalaCards database, and DisGeNET database. Then, we established the network concerning the interactions of potential targets of QBF with well-known psoriasis-related targets by using protein-protein interaction (PPI) data in String database. Afterwards, topological parameters (including DNMC, Degree, Closeness, and Betweenness) were calculated to excavate the core targets of Qubi Formula in treating psoriasis (main targets in the PPI network). Cytoscape was used to construct the ingredients-targets core network for Qubi Formula in treating psoriasis, and ClueGO was used to perform GO-BP and KEGG pathway enrichment analysis on these core targets.

**Results:**

The ingredient-target-disease core network of QBF in treating psoriasis was screened to contain 175 active ingredients, which corresponded to 27 core targets. Additionally, enrichment analysis suggested that targets of QBF in treating psoriasis were mainly clustered into multiple biological processes (associated with nuclear translocation of proteins, cellular response to multiple stimuli (immunoinflammatory factors, oxidative stress, and nutrient substance), lymphocyte activation, regulation of cyclase activity, cell-cell adhesion, and cell death) and related pathways (VEGF, JAK-STAT, TLRs, NF-*κ*B, and lymphocyte differentiation-related pathways), indicating the underlying mechanisms of QBF on psoriasis.

**Conclusion:**

In this work, we have successfully illuminated that Qubi Formula could relieve a wide variety of pathological factors (such as inflammatory infiltration and abnormal angiogenesis) of psoriasis in a “multicompound, multitarget, and multipathway” manner by using network pharmacology. Moreover, our present outcomes might shed light on the further clinical application of QBF on psoriasis treatment.

## 1. Introduction

Psoriasis is a common and frequently occurring disease in dermatology, which is characterized by easy diagnosis and difficult treatment, as well as recurrent disease course [1]. An epidemiological survey shows that the overall prevalence of psoriasis is approximately 0.5% in China [2]. The pathogenesis of psoriasis is still not completely clarified. Present studies have suggested that autoimmune disorders, dysfunction in various inflammatory signal transduction pathways, abnormal expression of psoriatic susceptibility gene, and obesity might be involved in the pathogenesis of psoriasis [3–9]. The unknown pathogenesis has brought difficulties to the treatment, without curative approaches against psoriasis at present. Traditional Chinese medicine (TCM) has unique advantages in treating psoriasis, which can play therapeutic roles through multiple targets and multiple pathways, corresponding to the dysfunction of various pathways underlying the pathogenesis of psoriasis [10–13]. However, TCM also has defects. Due to the unclear component entering the blood through the compound TCM, the mechanism of action is not completely clear, which restricts the further standardization and internationalization of TCM for psoriasis.

Qubi Formula (QBF) is an experience prescription for treating psoriasis at the Department of Dermatology in our hospital. It consists of Bubali Cornu (Shuiniujiao, SNJ, 30 g), Rehmanniae Radix (Dihuang, DH, 20 g), Paeoniae Radix Rubra (Chishao, CS, 10 g), Moutan Cortex (Mudanpi, MDP, 15 g), Arnebiae Radix (Zicao, ZC, 10 g), Lonicerae Japonicae Flos (Jinyinhua, JYH, 10 g), Forsythiae Fructus (Lianqiao, LQ, 10 g), Isatidis Radix (Banlangen, BLG, 30 g), and Glycyrrhizae Radix et Rhizoma (Gancao, GC, 6 g). QBF is modified from the classical TCM formula “Xi-Jiao-Di-Huang decoction.” In this formula, Bubali Cornu is used as the sovereign drug (Jun), while Rehmanniae Radix and Isatidis Radix are utilized as the minister herbs (Chen); Paeoniae Radix Rubra, Moutan Cortex, Arnebiae Radix, Lonicerae Japonicae Flos, and Forsythiae Fructus are the assistant herbs (Zuo), whereas Glycyrrhizae Radix et Rhizoma is the messenger herbs (Shi). The mixed application of these drugs exerts the effects of clearing away heat and removing toxicity, cooling blood, and receding speckles. The effects of QBF on psoriasis have been validated by clinical practice in multiple years, especially in patients with blood-heat subtypes of psoriasis. However, the scientific basis as well as potential pharmacological mechanisms of QBF is still unclear, which needs further investigations.

Conventional researches on the mechanism of one traditional Chinese medicine mostly follow the model of “one drug-one target-one disease,” which cannot reflect the characteristics of TCM (multicompound, multitarget, and multipathway). Herein, in this study, a comprehensive approach [14] (a combination of multiple network-based computational and algorithm-based approaches) was utilized, by combining prediction of active compounds based on multiple pharmacokinetic parameters, excavation of diverse drug targets, and network analysis from a macroscopic perspective, aiming at the illumination of the underlying mechanisms of QBF on psoriasis and providing ideas for subsequent research.

## 2. Materials and Methods

### 2.1. Screening of Potential Pharmacological Active Ingredients and Targets of Qubi Formula

BATMAN-TCM [15] (http://bionet.Ncpsb.Org/batman-tcm/index.Php/Home/Index/index) is a bioinformatics analysis tool for analyzing pharmacological active ingredients of Chinese medicines. In order to obtain the information about the ingredients of QBF, “SHUI NIU JIAO, DI HUANG, CHI SHAO, MU DAN PI, ZI CAO, JIN YIN HUA, LIAN QIAO, BAN LAN GEN, and GAN CAO” were used as key words to search in the BATMAN database, giving rise to a total of 572 compounds.

The Traditional Chinese Medicine Systems Pharmacology (TCMSP) database [16] (http://tcmspw.com/tcmsp.php) is a unique platform which can provide pharmacokinetic properties (involving oral bioavailability, drug-likeness, aqueous solubility, etc.) and potential targets for natural compounds. We searched the above 572 compounds in the TCMSP platform to obtain their pharmacokinetic parameters. After obtaining the pharmacokinetic information on the 572 compounds, the reference was screened according to oral bioavailability (OB) ≥30% and drug-likeness (DL) ≥0.18 (mean value for all molecules within the DrugBank database), aiming to screen potential pharmacologically active ingredients of QBF [17]. In this study, these cut-off values utilized helped to efficiently and maximally collect data from QBF using the least components, and the pharmacokinetic data reported may account for this [18]. OB [19], defined as the distribution degree of an oral dose of drug into bloodstream, is one of the most requisite premises in terms of oral drug discovery as well as clinical application. Additionally, drug-likeness, which is defined as a qualitative concept for assessment of the structural similarity of compounds with clinical therapeutics in the DrugBank database, is determined early after drug discovery [20]. In addition, through literature review, certain compounds with the OB < 30 or DL < 0.18 but with extensive pharmaceutical activities (such as oleic acid [21] and arnebinol [22]), or those with relatively higher contents (like jioglutin and alkannin) or those used for the quality identification of single herb in the Pharmacopoeia (goitrin), were also added as potential pharmacologically active ingredients of QBF.

Apart from assisting in exploring the active ingredients of TCM, TCMSP databases could also predict the potential targets of compounds based on SysDT model, HIT database, reverse molecular docking, etc. [23].

### 2.2. Collection of Known Psoriasis-Related Targets, Psoriasis

In order to obtain the known psoriasis-related targets, “psoriasis” was used as key word to search in Therapeutic Target Database (TTD) [24], DrugBank database [25], MalaCards database [26], and DisGeNET database [27]. After searching in the DisGeNET database, results were sorted by the disease specificity index (DSI), following by the removal of targets lower than the median of DSI obtained from all the known psoriasis-related genes. Additionally, the drugs with abnormal status in TTD and DrugBank database and their corresponding targets were also taken out. The detailed information of these known psoriasis-related was summarized in Table S1 after redundancy deletion.

### 2.3. Excavation of Core Targets of QBF for Treating Psoriasis and Construction of Core Network on Active Ingredients-Targets

First, targets obtained from the above two steps (potential targets of QBF and known psoriasis-related targets) were standardized in the UniProt database [28] by selecting the species “*Homo sapiens*,” aiming to acquire the single universal gene names. Then, both potential targets of QBF and known psoriasis-related targets were uploaded to the online Wayne diagram tool (http://bioinfogp.cnb.csic.es/tools/venny/index.html, Version 2.1.0) for mapping; that is, targets from these two sets were intersected to obtain the candidate targets of QBF for treating psoriasis.

Subsequently, the candidate targets were imported into the String database [29] to obtain protein-protein interactions (PPIs) by setting the minimum value of the combined score at 0.400 and the species as “*Homo sapiens*.” The topological parameters (DMNC, Degree, Closeness, and Betweenness) of each target (node) in the network were calculated using the cytoHubba plugin [30]. The median values of these four parameters of all nodes were used as a screening condition. Nodes with all the four parameter greater than the median values were considered as the main hubs that played core roles in the PPI network, that is, the core targets of QBF for psoriasis. Finally, Cytoscape was used to construct the core network of active ingredients-targets.

### 2.4. Enrichment Analysis of Core Targets

The ClueGO [31] plugin from Cytoscape software, integrative GO-biological process (BP), and KEGG database were applied to perform enrichment analysis on the core targets, and species was selected as “*Homo*” in the ClueGO interface. All the core targets were sequentially imported, followed by enrichment analysis. *κ* value was defaulted at 0.4 and *p* was set at ≤0.05, which were used as the screening conditions for plotting enrichment analysis.

## 3. Results

### 3.1. Screening of Potential Pharmacologically Active Ingredients and Their Targets of Qubi Formula

The search through the BATMAN-TCM database revealed a total of 572 compounds of QBF. Accumulative efforts have been made to clarify the therapeutic mechanisms of TCM, however, with sluggish progress on the molecular level. Due to the unavailable effective methods specifically developed for the identification of the active compounds in medicinal herbs, OB screening combined with drug-likeness assessment may be a feasible strategy. In this study, OB ≥ 30% and DL ≥ 0.18 were used as the screening conditions. Then, a total of 202 possible compounds with proper values of above two parameters were collected for potential pharmacologically active ingredients from the herbal constituents of QBF. In addition, among the compounds that were screened out, we found another 35 compounds through the literature research of PubMed. Although these 35 compounds did not meet the screening conditions of OB and DL, they were reported to use a wide range of pharmacological activities and were thus included in potential pharmacologically active ingredients. Finally, the active ingredients of SNJ, GC, CS, DH, MDP, ZC, JYH, LQ, and BLG were 6, 97, 26, 6, 12, 17, 30, 27, and 51, respectively. The proportions of the active ingredients in the sovereign drug (Jun), minister herbs (Chen), assistant herbs (Zuo), and messenger herbs (Shi) in all the 175 ingredients were 3.4%, 30.86%, 53.14%, and 55.43%, respectively. Among them, some compounds were widely present in multiple herbs of QBF, such as oleic acid, methyl linolenate, sitosterol, and paeoniflorin. The basic information of the potential pharmacologically active ingredients of Qubi Formula is shown in Table 1.

Subsequently, we explored the potential targets of the 237 potential pharmacologically active ingredients by excavating TCMSP databases, which yielded to 939 targets (shown in Table S2). The numbers of potential targets linked by SNJ, GC, CS, DH, MDP, ZC, JYH, LQ, and BLG were 668, 234, 148, 49, 207, 90, 219, 229, and 111, respectively. The proportions of the potential targets of the sovereign drug (Jun), minister herbs (Chen), assistant herbs (Zuo), and messenger herbs (Shi) in all the 939 targets were 71.14%, 11.93%, 30.35%, and 24.92%, respectively. Although the number of targets correlated with each herb of QBF is different, significant overlaps were observed in the nine herbs, which was suggestive of the congenerous or antergic roles of the various components in QBF via the regulation of similar targets.

**Figure 1:**
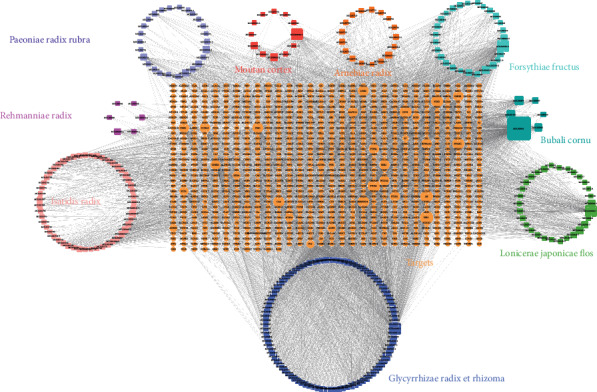
Construction of the QBF compound-potential target network. The compound-potential target network was constructed by linking the candidate compounds and their potential targets of the 9 herbs, which are constituents of QBF. The nodes representing candidate compounds are shown as polychrome square and the targets are indicated by orange circle.

In order to holistically and systemically obtain comprehensive understanding of the ingredient-target network in QBF, a network map was constructed by using Cytoscape, including 6273 edges and 1212 nodes (Figure 1). To be specific, the node degree indicated the number of target or edge correlated with the node according to topological analysis. A total of 142 ingredients were found in the as-established network to have the median of ≥18 degrees. Of them, quercetin, arginine, and oleic acids kaempferol and luteolin acted on 166, 96, and 54 targets, respectively, which were subsequently considered as the critical pharmacologically active ingredients of QBF.

### 3.2. Excavation of the Core Targets of Qubi Formula in Treating Psoriasis

**Figure 2:**
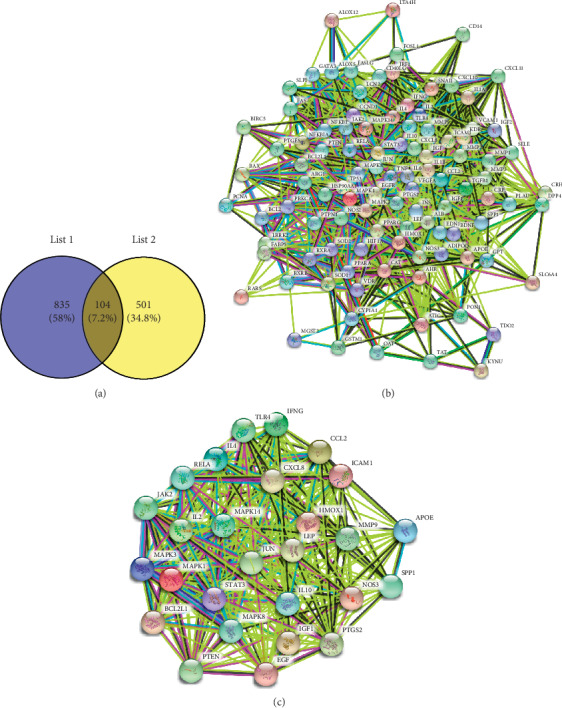
Excavation of the core target of QBF in treating psoriasis. (a) The Venn diagram showed that QBF shared 104 potential targets with known pathological course-related targets of psoriasis. (b) The PPI network of all the candidate targets of QBF in treating psoriasis. (c) The PPI of the core target of QBF in treating psoriasis.

Psoriasis has been recognized as the polygenic disorder. In addition, the investigation of the interactions between genes as well as gene and environment could be used to reveal the pathogenesis of psoriasis. After the targets with abnormal status from TTD and DrugBank database and DSI < 0.535 (the median of DSI) from DisGeNET database were excluded, we collected 605 targets (Table S1) associated with psoriasis from the four accessible resources. Notably, 104 of the identified potential targets of the QBF were also the well-recognized psoriasis disease- (or therapeutic drugs) related targets (Table S3 and Figure 2(a)). And these 104 targets were defined as the candidate targets for QBF in treating psoriasis.

Subsequently, to further select the core targets of QBF in treating psoriasis, the String database was used to construct the PPI network of the above targets (Figure 2(b)), followed by the calculation of the topological parameters (DMNC, Degree, Closeness, and Betweenness) of each node in the network using the cytoHubba plugin. The median values of these four parameters of all nodes were used as a screening condition. Nodes with all the four parameters greater than the median values were considered as the main hubs that played core roles in the PPI network. As a result, 27 targets (Table 2 and Table S4) were screened from the 104 candidate targets based on the values of topological parameters (Figure 2(c)), that is the core targets of QBF in treating psoriasis.

### 3.3. Construction of Active Ingredients-Targets Core Network for Qubi Formula in Treating Psoriasis

**Figure 3:**
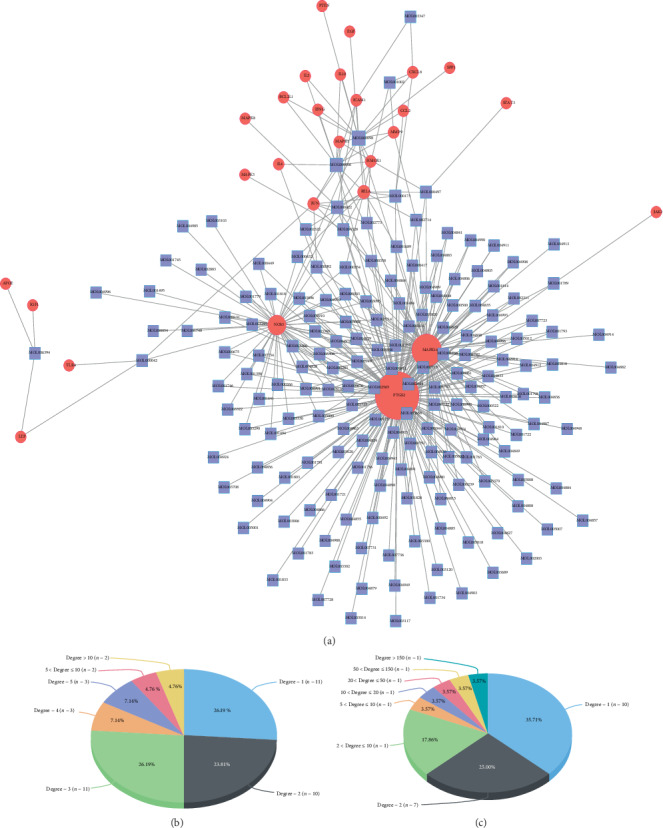
Construction of active ingredients-targets core network for QBF in treating psoriasis (a), and the statistical analysis of the degree of each ingredient (b) and target (c) in the network. All nodes were sorted and calculated according to the degree of freedom, and the node size in the network was associated with degree.

In order to further understand the “multicompound and multitarget” mechanism of Qubi Formula in treating psoriasis, we searched for the potential ingredients of Qubi Formula which could affect the 27 core targets based on the relationship between the ingredients and their targets, followed by construction of the core network on active ingredients-targets (Figure 3(a)) using Cytoscape software and the statistical analysis of the degree of each node in the network. As shown in Figure 3(b), the degrees of active ingredients ranged from 1 to 18 in the core network, with the median of 2, indicating that more than half of the compounds acted on more than one target, while the degree of the target ranged from 1 to 160, with the median of 2. Among all the active ingredients, the top 4 were quercetin, luteolin, kaempferol, and wogonin in terms of Degree. Previous preclinical studies have confirmed that all of them could delay the progression of psoriasis and relieve symptoms in multiple animal models [32–34]. Among all the targets, the top three were PTGS2, MAPK14, and NOS3 in terms of Degree, and all of them have been demonstrated to play important roles in the pathogenesis of psoriasis, such as inflammatory infiltration, abnormal differentiation of keratinocytes, and oxidative stress injury [35, 36].

### 3.4. Enrichment Analysis of the Core Targets of Qubi Formula in Treating Psoriasis

**Figure 4:**
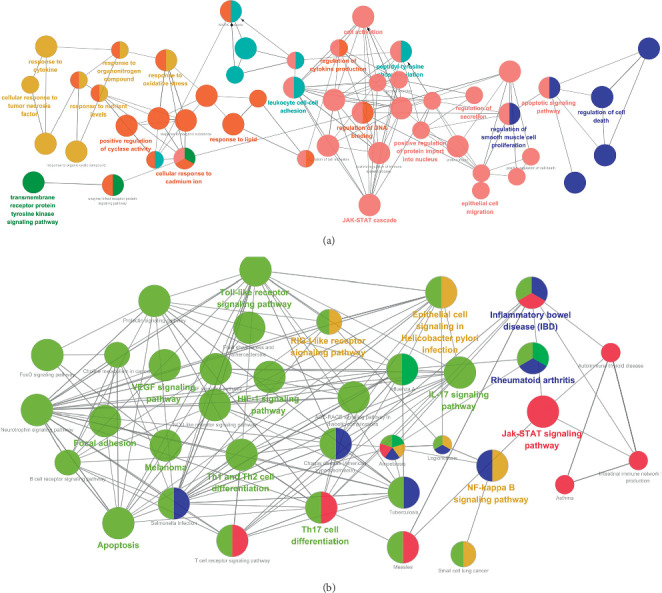
Enrichment analysis of candidate targets for QBF against psoriasis. The enrichment analysis is generated by ClueGO and the most vital term in the group is labeled. Functionally related groups partially overlap. Representative enriched biological process or pathway (*P* < 0.05) interactions among core QBF targets. The larger circle indicated the greater degree of enrichment, and the closer color suggested the more similar function in biological network. (a) Core QBF targets enriched in the representative biological process. (b) Core QBF targets enriched in the representative signaling pathway.

In order to further understand the mechanism of “multitarget and multipathway” of Qubi Formula in treating psoriasis, ClueGO plugin was used to perform enrichment analysis of GO-PB and KEGG on core targets and to excavate the biological processes and signaling pathways regulated by Qubi Formula in treating psoriasis. These 27 core targets were involved in several biological process, mainly including nuclear translocation of proteins, cellular response to multiple stimuli (immunoinflammatory factors, oxidative stress, and nutrient substance), lymphocyte activation, regulation of cyclase activity, cell-cell adhesion, and cell death (Figure 4(a)). Moreover, according to the *p*values of enriched pathways and their correlation with psoriasis, we were most interested in the following five representative signal pathways including VEGF, JAK-STAT, TLRs, NF-*κ*B, and lymphocyte differentiation-related pathway (Figure 4(b) and Table 3).

## 4. Discussion

Qubi Formula is an experience prescription for psoriasis at the Department of Dermatology in our hospital. It is especially suitable for patients with blood-heat subtype of psoriasis, with radiated skin lesions throughout the whole body, redness, obvious scales, itching and burning, red tongue, and yellow fur. In this formula, SNJ, DH, JYH, and BLG can clear heat, cool blood, and remove toxic materials. MDP and CS are responsible for cooling blood and removing blood stasis. GC is in charge of detoxifying and reconciling medicine. The therapeutic effects of Qubi Formula in the clinical treatment of psoriasis are significant. However, the active ingredients and potential targets of Qubi Formula are unclear, which hinders the further development and application of the prescription.

Network pharmacology is a new strategy for drug design and development based on the rapid development of systematical biology and multidirectional pharmacology. This concept was first proposed by Hopkins AL in 2007, which was switched from previous “disease-single target-single drug” model of new drug development to the “disease-multitarget-multidrug” model. This idea coincides with the “holistic view” of TCM. Therefore, the application of the network pharmacology method can provide certain research ideas for discovering the mechanism of Qubi Formula in treating psoriasis.

In this study, a total of 175 potential active ingredients of Qubi Formula in treating psoriasis were screened through a series of network pharmacological methods, which corresponded to 27 core targets. At present, the widely acknowledged histopathological features of psoriasis include four major aspects: the inflammatory infiltration in dermis and epidermis, the abnormal biological behaviours (differentiation, hyperproliferation, and apoptosis) of keratinocytes, metabolic disturbance in skin tissue, and the tortuously increased dermal blood vessels and capillaries [37–41]. Firstly, among the 27 core targets, most of them (PTGS2, ILs, JAK2, STAT3, RELA, CCL2, CXCL8, EGF, IFNG, and TLR4) have been shown to be involved in abnormal inflammatory infiltration, which could regulate the differentiation and chemotaxis of lymphocytes, cytokines produced, and immunological inflammatory reaction in dermis and epidermis [42–48]. Secondly, RELA, MAPKs, JUN, and BCL2L1 are associated with the aberrant biological behaviours of keratinocytes in psoriasis [49–51]. Thirdly, APOE, HMOX1, LEP, IGF1, and SPP1 have been demonstrated to take part in metabolic disturbance (including lipids, peroxides, and carbohydrates) in skin tissue [52–54]. Finally, ICAM1, MM9, and NOS3 are closely associated with endothelial cell proliferation, migration, and adhesion, which are related to the tortuously increased dermal blood vessels and capillaries [55, 56].

Enrichment analysis of GO-BP and KEGG on the core targets further suggests that Qubi Formula could intervene in psoriasis through multiple biological processes by acting on several signaling pathways, involving VEGF, JAK-STAT, TLRs, NF-*κ*B, and lymphocyte differentiation-related pathways. As shown in the Figure 4(a), these five signaling pathways cross-talk effects in the network. VEGF signal pathway has been demonstrated to not only induce pathological angiogenesis in psoriatic lesions by regulating the proliferation and differentiation of endothelial cells, but also aggravate the inflammatory response via increasing vascular permeability to promote inflammatory cell infiltration [57, 58]. Moreover, as it is well known that psoriasis is an inflammatory disease mediated by T lymphocytes, abnormal differentiation of T lymphocytes (especially Th1 and Th17 cells) and excessive secretion of proinflammatory factors (such as ILs) are closely related to the progression of the disease [59]. In this study, we have also demonstrated that there are diverse critical signaling pathways related to T lymphocytes differentiation and proinflammatory factors production (TLRs, JAK-STAT, and NF-*κ*B) regulated by QBF on psoriasis therapy [60–62].

Our team has previously confirmed the safety and effectiveness of Qubi Formula in treating psoriasis through clinical observations. Based on the results of this study, we speculate that the regulatory role of Qubi Formula on psoriasis is not unilateral, but is directly or indirectly involved in the comprehensive treatment of the four major pathological factors of psoriasis through multiple signaling pathways associated with immunoinflammatory response, metabolism, and abnormal angiogenesis. Despite the valuable discoveries, there are still certain limitations. In the present study concerning the network pharmacological analysis on Qubi Formula, only the interactions between the components of QBF and the psoriasis-related targets were considered, but the interactions between the ingredients, the dosage of each ingredient, and the effects of the different processing methods of medicinal materials were neglected. Therefore, the obtained results must be verified by further experiments.

## 5. Conclusion

The present study illustrates the systemic “multicompound and multitarget” efficacy of QBF against psoriasis. Moreover, this study also provided a theoretical basis to determine the synergistic effects of TCM in treating diseases and the role of systematic network pharmacology in elucidating the potential mechanisms of action of TCMs. However, as this study was based on data mining and data analysis, further validation studies should be undertaken.

## Figures and Tables

**Table 1:** All the potential pharmacologically active ingredients of QBF.

Herb name	Molecule ID	Molecule name	OB (%)	DL
Bubali Cornu (Shuiniujiao, SNJ)	MOL000054	Arginine	47.64	0.03
	MOL000065	Aspartic acid	79.74	0.02
	MOL000042	Alanine	87.69	0.01
	MOL001443	4-Guanidino-1-butanol	26.23	0.01
	MOL006394	Guanidine	24	0
	MOL000987	Cholesterol	37.87	0.68

Glycyrrhizae Radix et Rhizoma (Gancao, GC)	MOL000057	DIBP	49.63	0.13
	MOL000098	Quercetin	46.43	0.28
	MOL000211	Mairin	55.38	0.78
	MOL000239	Jaranol	50.83	0.29
	MOL000354	Isorhamnetin	49.6	0.31
	MOL000359	Sitosterol	36.91	0.75
	MOL000392	Formononetin	69.67	0.21
	MOL000417	Calycosin	47.75	0.24
	MOL000422	Kaempferol	41.88	0.24
	MOL000497	Licochalcone a	40.79	0.29
	MOL000500	Vestitol	74.66	0.21
	MOL000676	DBP	64.54	0.13
	MOL001484	Inermine	75.18	0.54
	MOL001792	DFV	32.76	0.18
	MOL002311	Glycyrol	90.78	0.67
	MOL002565	Medicarpin	49.22	0.34
	MOL002844	Pinocembrin	64.72	0.18
	MOL003656	Lupiwighteone	51.64	0.37
	MOL003896	7-Methoxy-2-methyl isoflavone	42.56	0.2
	MOL004328	Naringenin	59.29	0.21
	MOL004805	(2S)-2-[4-Hydroxy-3-(3-methylbut-2-enyl)phenyl]-8,8-dimethyl-2,3-dihydropyrano[2,3-f]chromen-4-one	31.79	0.72
	MOL004806	Euchrenone	30.29	0.57
	MOL004808	Glyasperin B	65.22	0.44
	MOL004810	Glyasperin F	75.84	0.54
	MOL004811	Glyasperin C	45.56	0.4
	MOL004814	Isotrifoliol	31.94	0.42
	MOL004815	(E)-1-(2,4-Dihydroxyphenyl)-3-(2,2-dimethylchromen-6-yl)prop-2-en-1-one	39.62	0.35
	MOL004820	Kanzonol W	50.48	0.52
	MOL004824	(2S)-6-(2,4-Dihydroxyphenyl)-2-(2-hydroxypropan-2-yl)-4-methoxy-2,3-dihydrofuro[3,2-g]chromen-7-one	60.25	0.63
	MOL004827	Semilicoisoflavone B	48.78	0.55
	MOL004828	Glepidotin A	44.72	0.35
	MOL004829	Glepidotin B	64.46	0.34
	MOL004833	Phaseolinisoflavan	32.01	0.45
	MOL004835	Glypallichalcone	61.6	0.19
	MOL004836	Echinatin	66.58	0.17
	MOL004838	8-(6-Hydroxy-2-benzofuranyl)-2,2-dimethyl-5-chromenol	58.44	0.38
	MOL004841	Licochalcone B	76.76	0.19
	MOL004848	Licochalcone G	49.25	0.32
	MOL004849	3-(2,4-Dihydroxyphenyl)-8-(1,1-dimethylprop-2-enyl)-7-hydroxy-5-methoxy-coumarin	59.62	0.43
	MOL004855	Licoricone	63.58	0.47
	MOL004856	Gancaonin A	51.08	0.4
	MOL004857	Gancaonin B	48.79	0.45
	MOL004860	Licorice glycoside E	32.89	0.27
	MOL004863	3-(3,4-Dihydroxyphenyl)-5,7-dihydroxy-8-(3-methylbut-2-enyl)chromone	66.37	0.41
	MOL004864	5,7-Dihydroxy-3-(4-methoxyphenyl)-8-(3-methylbut-2-enyl)chromone	30.49	0.41
	MOL004866	2-(3,4-Dihydroxyphenyl)-5,7-dihydroxy-6-(3-methylbut-2-enyl)chromone	44.15	0.41
	MOL004879	Glycyrin	52.61	0.47
	MOL004882	Licocoumarone	33.21	0.36
	MOL004883	Licoisoflavone	41.61	0.42
	MOL004884	Licoisoflavone B	38.93	0.55
	MOL004885	Licoisoflavanone	52.47	0.54
	MOL004891	Shinpterocarpin	80.3	0.73
	MOL004898	(E)-3-[3,4-Dihydroxy-5-(3-methylbut-2-enyl)phenyl]-1-(2,4-dihydroxyphenyl)prop-2-en-1-one	46.27	0.31
	MOL004903	Liquiritin	65.69	0.74
	MOL004904	Licopyranocoumarin	80.36	0.65
	MOL004905	3,22-Dihydroxy-11-oxo-delta(12)-oleanene-27-alpha-methoxycarbonyl-29-oic acid	34.32	0.55
	MOL004907	Glyzaglabrin	61.07	0.35
	MOL004908	Glabridin	53.25	0.47
	MOL004910	Glabranin	52.9	0.31
	MOL004911	Glabrene	46.27	0.44
	MOL004912	Glabrone	52.51	0.5
	MOL004913	1,3-Dihydroxy-9-methoxy-6-benzofurano[3,2-c]chromenone	48.14	0.43
	MOL004914	1,3-Dihydroxy-8,9-dimethoxy-6-benzofurano[3,2-c]chromenone	62.9	0.53
	MOL004915	Eurycarpin A	43.28	0.37
	MOL004917	Glycyroside	37.25	0.79
	MOL004924	(−)-Medicocarpin	40.99	0.95
	MOL004935	Sigmoidin-B	34.88	0.41
	MOL004941	(2R)-7-Hydroxy-2-(4-hydroxyphenyl)chroman-4-one	71.12	0.18
	MOL004945	(2S)-7-Hydroxy-2-(4-hydroxyphenyl)-8-(3-methylbut-2-enyl)chroman-4-one	36.57	0.32
	MOL004948	Isoglycyrol	44.7	0.84
	MOL004949	Isolicoflavonol	45.17	0.42
	MOL004957	HMO	38.37	0.21
	MOL004959	1-Methoxyphaseollidin	69.98	0.64
	MOL004961	Quercetin der.	46.45	0.33
	MOL004964	(Z)-1-(2,4-Dihydroxyphenyl)-3-phenylprop-2-en-1-one	73.18	0.12
	MOL004966	3′-Hydroxy-4′-O-Methylglabridin	43.71	0.57
	MOL004974	3′-Methoxyglabridin	46.16	0.57
	MOL004978	2-[(3R)-8,8-Dimethyl-3,4-dihydro-2H-pyrano[6,5-f]chromen-3-yl]-5-methoxyphenol	36.21	0.52
	MOL004980	Inflacoumarin A	39.71	0.33
	MOL004985	Icos-5-enoic acid	30.7	0.2
	MOL004988	Kanzonol F	32.47	0.89
	MOL004989	6-Prenylated eriodictyol	39.22	0.41
	MOL004990	7,2′,4′-Trihydroxy-5-methoxy-3-arylcoumarin	83.71	0.27
	MOL004991	7-Acetoxy-2-methylisoflavone	38.92	0.26
	MOL004993	8-Prenylated eriodictyol	53.79	0.4
	MOL004996	Gadelaidic acid	30.7	0.2
	MOL005000	Gancaonin G	60.44	0.39
	MOL005001	Gancaonin H	50.1	0.78
	MOL005003	Licoagrocarpin	58.81	0.58
	MOL005007	Glyasperin M	72.67	0.59
	MOL005008	*Glycyrrhiza* flavonol A	41.28	0.6
	MOL005012	Licoagroisoflavone	57.28	0.49
	MOL005013	18*α*-Hydroxyglycyrrhetic acid	41.16	0.71
	MOL005016	Odoratin	49.95	0.3
	MOL005017	Phaseol	78.77	0.58
	MOL005018	Xambioona	54.85	0.87
	MOL005020	Dehydroglyasperin C	53.82	0.37

Paeoniae Radix Rubra (Chishao, CS)	MOL001924	Paeoniflorin	53.87	0.79
	MOL000449	Stigmasterol	43.83	0.76
	MOL004355	Spinasterol	42.98	0.76
	MOL000358	Beta-sitosterol	36.91	0.75
	MOL000359	Sitosterol	36.91	0.75
	MOL002776	Baicalin	40.12	0.75
	MOL006999	Stigmast-7-en-3-ol	37.42	0.75
	MOL005043	Campest-5-en-3beta-ol	37.58	0.71
	MOL007003	Benzoyl paeoniflorin	31.14	0.54
	MOL007025	Isobenzoyl paeoniflorin	31.14	0.54
	MOL001002	Ellagic acid	43.06	0.43
	MOL001918	Paeoniflorgenone	87.59	0.37
	MOL007016	Paeoniflorigenone	65.33	0.37
	MOL006996	1-o-Beta-d-glucopyranosylpaeonisuffrone_qt	65.08	0.35
	MOL007005	Albiflorin_qt	48.7	0.33
	MOL006992	(2R,3 R)-4-Methoxyl-distylin	59.98	0.3
	MOL006994	1-o-Beta-d-glucopyranosyl-8-o-benzoylpaeonisuffrone_qt	36.01	0.3
	MOL007018	9-Ethyl-neo-paeoniaflorin A_qt	64.42	0.3
	MOL006990	(1S,2S,4R)-trans-2-hydroxy-1,8-cineole-B-D-glucopyranoside	30.25	0.27
	MOL000492	(+)-catechin	54.83	0.24
	MOL002714	Baicalein	33.52	0.21
	MOL002883	Ethyl oleate (NF)	32.4	0.19
	MOL001641	Methyl linoleate	41.93	0.17
	MOL000131	EIC	41.9	0.14
	MOL000675	Oleic acid	33.13	0.14
	MOL001746	ELD	31.2	0.14

Rehmanniae Radix (Dihuang, DH)	MOL000449	Stigmasterol	43.83	0.76
	MOL000359	Sitosterol	36.91	0.75
	MOL000131	EIC	41.9	0.14
	MOL003708	Jioglutin D	39.02	0.14
	MOL003689	Aeginetic acid	48.31	0.13
	MOL003706	Jioglutin B	90.71	0.13

Moutan Cortex (Mudanpi, MDP)	MOL000211	Mairin	55.38	0.78
	MOL000359	Sitosterol	36.91	0.75
	MOL007003	Benzoyl paeoniflorin	31.14	0.54
	MOL007369	4-O-methylpaeoniflorin_qt	67.24	0.43
	MOL001925	Paeoniflorin_qt	68.18	0.4
	MOL007382	Mudanpioside-h_qt 2	42.36	0.37
	MOL007384	Paeonidanin_qt	65.31	0.35
	MOL007374	5-[[5-(4-Methoxyphenyl)-2-furyl]methylene]barbituric acid	43.44	0.3
	MOL000098	Quercetin	46.43	0.28
	MOL000422	Kaempferol	41.88	0.24
	MOL000492	(+)-Catechin	54.83	0.24
	MOL000675	Oleic acid	33.13	0.14

Arnebiae Radix (Zicao, ZC)	MOL000359	Sitosterol	36.91	0.75
	MOL002372	(6Z,10 E,14E,18 E)-2,6,10,15,19,23-Hexamethyltetracosa-2,6,10,14,18,22-hexaene	33.55	0.42
	MOL007736	Lithospermidin B	60.48	0.39
	MOL007728	Lithospermidin A	75.08	0.38
	MOL007714	1-Methoxyacetylshikonin	73.09	0.29
	MOL007715	[(1R)-1-(5,8-Dihydroxy-1,4-dioxo-2-naphthyl)-4-methyl-pent-3-enyl] propanoate	54.64	0.29
	MOL007716	Acetylshikonin	62.39	0.27
	MOL007734	5-[(E)-5-(3-Furyl)-2-methyl-pent-2-enyl]-2,3-dimethoxy-p-benzoquinone	61.8	0.24
	MOL007722	Isoarnebin 4	64.79	0.2
	MOL007735	Des-O-methyllasiodiplodin	30.12	0.2
	MOL001494	Mandenol	42	0.19
	MOL002883	Ethyl oleate (NF)	32.4	0.19
	MOL007719	Arnebin 7	73.85	0.18
	MOL007717	Alkannin	6.09	0.35
	MOL000131	EIC	41.9	0.14
	MOL000675	Oleic acid	33.13	0.14
	MOL007731	Arnebinol	56.66	0.14

Lonicerae Japonicae Flos (Jinyinhua, JYH)	MOL003036	(3S,8 R,9R,10 R,13R,14S,17R)-17-[(E,2R,5S)-5-Ethyl-6-methylhept-3-en-2-yl]-10,13-dimethyl-2,3,4,7,8,9,11,12,14,15,16,17-dodecahydro-1H-cyclopenta[a]phenanthren-3-ol	43.83	0.76
	MOL000449	Stigmasterol	43.83	0.76
	MOL000358	Beta-sitosterol	36.91	0.75
	MOL003108	Caeruloside C	55.64	0.73
	MOL003124	Xylostosidine	43.17	0.64
	MOL002773	Beta-carotene	37.18	0.58
	MOL003101	7-Epivogeloside	46.13	0.58
	MOL003059	Kryptoxanthin	47.25	0.57
	MOL003062	4,5′-Retro-.beta.,.beta.-carotene-3,3′-dione, 4′,5′-didehydro-	31.22	0.55
	MOL002707	Phytofluene	43.18	0.5
	MOL003111	Centauroside_qt	55.79	0.5
	MOL003128	Dinethylsecologanoside	48.46	0.48
	MOL003095	5-Hydroxy-7-methoxy-2-(3,4,5-trimethoxyphenyl)chromone	51.96	0.41
	MOL003014	Secologanic dibutylacetal_qt	53.65	0.29
	MOL000098	Quercetin	46.43	0.28
	MOL003044	Chrysoeriol	35.85	0.27
	MOL000006	Luteolin	36.16	0.25
	MOL002914	Eriodyctiol (flavanone)	41.35	0.24
	MOL000422	Kaempferol	41.88	0.24
	MOL003006	(-)-(3R,8S,9R,9aS,10aS)-9-Ethenyl-8-(beta-D-glucopyranosyloxy)-2,3,9,9a,10,10a-hexahydro-5-oxo-5H,8H-pyrano[4,3-d]oxazolo[3,2-a]pyridine-3-carboxylic acid_qt	87.47	0.23
	MOL001495	Ethyl linolenate	46.1	0.2
	MOL001494	Mandenol	42	0.19
	MOL003117	Ioniceracetalide B_qt	61.19	0.19
	MOL001398	Methyl linolenate	46.15	0.17
	MOL001641	Methyl linoleate	41.93	0.17
	MOL003103	Methyl octadeca-8,11-dienoate	41.93	0.17
	MOL003120	Loniceracetalide A_qt	89.38	0.17
	MOL002003	(-)-Caryophyllene oxide	32.67	0.13
	MOL000266	Beta-cubebene	32.81	0.11
	MOL002697	Junipene	44.07	0.11

Forsythiae Fructus (Lianqiao, LQ)	MOL000791	Bicuculline	69.67	0.88
	MOL003305	Phillyrin	36.4	0.86
	MOL003365	Lactucasterol	40.99	0.85
	MOL000522	Arctiin	34.45	0.84
	MOL003281	20(S)-Dammar-24-ene-3*β*,20-diol-3-acetate	40.23	0.82
	MOL003315	3beta-Acetyl-20,25-epoxydammarane-24alpha-ol	33.07	0.79
	MOL000211	Mairin	55.38	0.78
	MOL000358	Beta-sitosterol	36.91	0.75
	MOL003344	*β*-Amyrin acetate	42.06	0.74
	MOL003348	Adhyperforin	44.03	0.61
	MOL003347	Hyperforin	44.03	0.6
	MOL003295	(+)-Pinoresinol monomethyl ether	53.08	0.57
	MOL003306	ACon1_001697	85.12	0.57
	MOL003308	(+)-Pinoresinol monomethyl ether-4-D-beta-glucoside_qt	61.2	0.57
	MOL003322	Forsythinol	81.25	0.57
	MOL003330	(−)-Phillygenin	95.04	0.57
	MOL003290	(3R,4 R)-3,4-bis[(3,4-Dimethoxyphenyl)methyl]oxolan-2-one	52.3	0.48
	MOL003283	(2R,3 R,4S)-4-(4-Hydroxy-3-methoxy-phenyl)-7-methoxy-2,3-dimethylol-tetralin-6-ol	66.51	0.39
	MOL003370	Onjixanthone I	79.16	0.3
	MOL000098	Quercetin	46.43	0.28
	MOL000006	Luteolin	36.16	0.25
	MOL000422	Kaempferol	41.88	0.24
	MOL000173	Wogonin	30.68	0.23
	MOL003358	Euxanthone	92.98	0.16
	MOL003302	Forsythidmethylester_qt	121.84	0.12
	MOL003360	Norlapachol	46.99	0.11
	MOL003300	Forsythide_qt	46.6	0.1

Isatidis Radix (Banlangen, BLG)	MOL001810	6-(3-Oxoindolin-2-ylidene)indolo[2,1-b]quinazolin-12-one	45.28	0.89
	MOL001806	Stigmasta-5,22-diene-3beta,7beta-diol	42.56	0.83
	MOL001804	Stigmasta-5,22-diene-3beta,7alpha-diol	43.04	0.82
	MOL001755	24-Ethylcholest-4-en-3-one	36.08	0.76
	MOL000449	Stigmasterol	43.83	0.76
	MOL001771	Poriferast-5-en-3beta-ol	36.91	0.75
	MOL001800	Rosasterol	35.87	0.75
	MOL000358	Beta-sitosterol	36.91	0.75
	MOL000359	Sitosterol	36.91	0.75
	MOL002322	Isovitexin	31.29	0.72
	MOL001790	Linarin	39.84	0.71
	MOL000953	CLR	37.87	0.68
	MOL001769	Beta-sitosterol decantate	34.57	0.57
	MOL001783	2-(9-((3-Methyl-2-oxopent-3-en-1-yl)oxy)-2-oxo-1,2,8,9-tetrahydrofuro[2,3-h]quinolin-8-yl)propan-2-yl acetate	64	0.57
	MOL001828	3-[(3,5-Dimethoxy-4-oxo-1-cyclohexa-2,5-dienylidene)methyl]-2,4-dihydro-1H-pyrrolo[2,1-b]quinazolin-9-one	51.84	0.56
	MOL001811	Goitrin	3.23	0.01
	MOL001750	Glucobrassicin	66.02	0.48
	MOL001734	3-[[(2R,3 R,5R,6S)-3,5-Dihydroxy-6-(1H-indol-3-yloxy)-4-oxooxan-2-yl]methoxy]-3-oxopropanoic acid	85.87	0.47
	MOL001779	Sinoacutine	49.11	0.46
	MOL001803	Sinensetin	50.56	0.45
	MOL001721	Isaindigodione	60.12	0.41
	MOL001733	Eupatorin	30.23	0.37
	MOL001749	ZINC03860434	43.59	0.35
	MOL001793	(E)-2-[(3-Indole)cyanomethylene-]-3-indolinone	54.59	0.32
	MOL001722	2-O-beta-D-Glucopyranosyl-2H-1,4-benzoxazin-3(4H)-one	43.62	0.31
	MOL001767	Hydroxyindirubin	63.37	0.3
	MOL001774	Ineketone	37.14	0.3
	MOL001735	Dinatin	30.97	0.27
	MOL001736	(-)-Taxifolin	60.51	0.27
	MOL001798	Neohesperidin_qt	71.17	0.27
	MOL001781	Indigo	38.2	0.26
	MOL001782	(2Z)-2-(2-Oxoindolin-3-ylidene)indolin-3-one	48.4	0.26
	MOL001814	(E)-3-(3,5-Dimethoxy-4-hydroxy-benzylidene)-2-indolinone	57.18	0.25
	MOL001820	(E)-3-(3,5-Dimethoxy-4-hydroxyb-enzylidene)-2-indolinone	65.17	0.25
	MOL001689	Acacetin	34.97	0.24
	MOL001833	Glucobrassicin-1-sulfonate_qt	42.52	0.24
	MOL001756	Quindoline	33.17	0.22
	MOL001728	3-[ 2′-(5′-Hydroxymethyl)furyl]-1(2H)-isoquinolinone-7-O-beta-D-glucoside_qt	51.74	0.18
	MOL001792	DFV	32.76	0.18
	MOL001398	Methyl linolenate	46.15	0.17
	MOL001745	Methyl vaccenate	31.9	0.17
	MOL001748	Methyl (E)-octadec-8-enoate	31.9	0.17
	MOL001763	3-(2-Hydroxyphenyl)quinazolin-4-one	63.58	0.16
	MOL001789	Isoliquiritigenin	85.32	0.15
	MOL001821	Methyl 2-ethylhexyl phthalate	65.98	0.15
	MOL000432	Linolenic acid	45.01	0.15
	MOL000131	EIC	41.9	0.14
	MOL001746	ELD	31.2	0.14
	MOL000057	DIBP	49.63	0.13
	MOL000676	DBP	64.54	0.13
	MOL001818	Methyl palmitelaidate	34.61	0.12

**Table 2:** Topological feature values of all the core targets for QBF against psoriasis.

Node name	DMNC	Degree	Closeness	Betweenness
APOE	1.19	47.00	74.33	41.76
BCL2L1	1.19	49.00	75.33	46.09
CCL2	1.15	73.00	87.50	105.15
CXCL8	1.12	75.00	88.50	144.89
EGF	1.18	63.00	82.33	83.27
HMOX1	1.19	56.00	79.00	64.03
ICAM1	1.24	64.00	82.83	48.67
IFNG	1.21	62.00	81.83	59.30
IGF1	1.23	62.00	81.83	48.60
IL10	1.13	74.00	88.00	128.25
IL2	1.17	62.00	81.83	117.83
IL4	1.20	65.00	83.33	67.65
JAK2	1.24	48.00	74.67	47.23
JUN	1.15	74.00	88.00	111.79
LEP	1.17	59.00	80.50	68.01
MAPK1	1.14	67.00	84.33	135.31
MAPK14	1.23	56.00	78.83	41.89
MAPK3	1.12	73.00	87.33	164.81
MAPK8	1.14	71.00	86.50	114.31
MMP9	1.13	73.00	87.50	225.47
NOS3	1.21	55.00	78.33	50.16
PTEN	1.17	49.00	75.33	46.86
PTGS2	1.14	73.00	87.50	224.73
RELA	1.18	54.00	77.83	41.41
SPP1	1.25	51.00	76.50	39.41
STAT3	1.16	72.00	86.83	132.54
TLR4	1.12	72.00	87.00	135.66

**Table 3:** Representative enriched KEGG pathway of the core targets of Qubi Formula in treating psoriasis.

Pathway	Gene count	*P* value	Pathway ID	Associated genes
Th17 cell differentiation	11	3.87*E* − 12	ko04659	IFNG, IL2, IL4, JAK2, JUN, MAPK1, MAPK14, MAPK3, MAPK8, RELA, STAT3
Th1 and Th2 cell differentiation	10	4.32*E* − 11	ko04658	IFNG, IL2, IL4, JAK2, JUN, MAPK1, MAPK14, MAPK3, MAPK8, RELA
Toll-like receptor signaling pathway	9	7.14*E* − 11	ko04620	JUN, MAPK1, MAPK14, MAPK3, MAPK8, RELA, CXCL8, SPP1, TLR4
JAK-STAT signaling pathway	9	1.20*E* − 09	ko04630	IL2, IL4, JAK2, STAT3, BCL2L1, EGF, IL10, LEP, IFNG
VEGF signaling pathway	5	1.14*E* − 06	ko04370	MAPK1, MAPK14, MAPK3, NOS3, PTGS2
NF-kappa B signaling pathway	6	2.24*E* − 06	ko04064	PTGS2, BCL2L1, RELA, CXCL8, TLR4, ICAM1

## Data Availability

The data used to support the findings of this study are available from the corresponding author upon request.

## Supplementary Materials

Supplementary Materials
*Table S1*. Known psoriasis-related targets. *Table S2*. All the potential targets of QBF. *Table S3*. QBF shared 104 potential targets with known psoriasis-related targets. *Table S4*. Topological feature values of all the candidate targets for QBF against psoriasis.
